# Surgical treatment of upper cervical spine metastases: a retrospective study of 39 cases

**DOI:** 10.1186/s12957-016-1085-0

**Published:** 2017-01-14

**Authors:** Jian Yang, Qi Jia, Dongyu Peng, Wei Wan, Nanzhe Zhong, Yan Lou, Xiaopan Cai, Zhipeng Wu, Chenglong Zhao, Xinghai Yang, Jianru Xiao

**Affiliations:** Department of Orthopedic Oncology, Changzheng Hospital, Second Military Medical University, 415 Fengyang Road, Shanghai, 200003 China

**Keywords:** Upper cervical spine, Metastasis, Surgery, Reconstruction, Prognosis

## Abstract

**Background:**

The surgical treatment of upper cervical spine metastases are controversial up to now. By summarizing and analyzing the clinical data of the upper cervical spine involved metastases treated surgically in our center, we mainly aimed to investigate the surgical decisions and outcomes so as to provide more references for the clinical treatment of this special and complex spine metastasis.

**Methods:**

We evaluated the patients’ pre- and post-operative neck pain and neurologic function with paired *t* test, followed by the statistics of the selection of surgical approaches, ways of reconstruction, and related complications. Moreover, the Kaplan–Meier survival analysis was adopted to analyze the patients’ survival according to different growth group (rapid, moderate, and slow).

**Results:**

There were 39 patients with atlantoaxial metastases in this study. The most common symptom (94.87%) was occipital-cervical pain, which relieved greatly after surgical interventions (*p* < 0.01). The metastases mainly resulted from lung cancer and nasopharyngeal cancer with an incidence of 38.46 and 10.26%, respectively. As to different growth group, the rapid-growth tumors accounted for 69.23% in all atlantoaxial metastases. Tumor resection and stabilization were performed mainly via the combined anterior and posterior approach (66.67%). The 1-, 2-, and 3-year overall survival rate at the last follow-up was 58.5, 40, and 28.3%, respectively, with a median survival time of 18 months. The rate of complications associated with the surgical intervention was 12.82% (5/39), which is lower than that of the previous reports and generally controllable.

**Conclusions:**

Relatively radical interventions with surgery for upper cervical spine metastases offered satisfactory outcomes with a low mortality. Together with adjuvant therapy, surgical treatment benefits patients with atlantoaxial metastases by relieving regional pain, restoring or improving the neurologic function, stabilizing the quality of life, and prolonging the survival time of such patients.

## Background

The spine is the most common location of bone metastasis with an incidence of 70% in terminal cancer patients [1], including 8–25% in the cervical spine [2], while only about 0.5% in the upper cervical spine [3]. Studies about upper cervical metastases treatments are limited to case reports or small series [4–12]. Atlantoaxial metastasis usually not only results in severe neck pain [1, 7–9, 11, 13–15] but also leads to serious consequences such as paralysis of high position and death [7–9, 11].

Atlantoaxial metastasis generally involve the anterior elements, which is similar to tumors of the subaxial cervical spine. Treatment options for atlantoaxial metastases have always been controversial and difficult due to the great difficulty in exposure, resection, and reconstruction as a result of the complex anatomies. Irradiation, halo vest or collar support, or posterior-only decompression, and stabilization are the common treatments for such metastases [6–11]. Due to the critical position of the upper cervical spine, progression of the tumors would occur and cord compression even life-threatening events could be inevitable [9, 11, 16]. Although conservative treatment has a limited effect in symptoms relief and prognosis [9, 11, 13, 17], studies about the attempt of tumor resection and reconstruction in patients with upper cervical spine metastasis are hardly found up to now [4, 12, 18].

In the present study, we retrospectively analyzed the clinical features, pathologies, and the approaches, reconstructions, and outcomes of surgical treatment for atlantoaxial metastases in an attempt to provide new references for the treatment of upper cervical metastases. To the best of our knowledge, this is the largest study that focuses on the surgical treatment of atlantoaxial metastasis.

## Methods

Among the 4158 patients of spinal tumor from 2002 to 2014, the patients who were treated surgically for metastatic involvement of atlas and/or axis in our center were retrospectively analyzed in this study (Table 1). Pain was assessed pre- and post-operatively using the visual analog scale (VAS), which has a score scale of 0–10. In addition, the neurologic function was assessed according to the Frankel grade. According to the results of PET-CT scan or emission computed tomography, the Tomita scoring system was used in all patients to evaluate the condition of metastasis at admission. Kaplan–Meier survival analysis was conducted in all the patients as well as in different (rapid, moderate, and slow) growth groups.

**Table 1:** Clinical data of 39 atlantoaxil metastases treated surgically in this study

Case	Age/sex	Primary tumor	Growth	Symptom	Level	Operation			Complication	Adjuvant therapy		Frankel	Follow-up (mos)	
						Approach	Resection	Reconstruction		Pre-operation	Post-operation		Survival status	
1	55/F	Lung	Rapid	Pain	C1	HCA + P	Piecemeal	OPCF	None	C	R	E/E	5	Death
2	55/M	Lung	Rapid	Pain	C1–2	HCA + P	Piecemeal	OPCF	None	—	C	D/E	33	Death
3	65/M	Lung	Rapid	Pain	C2–3	HCA + P	Subtotal	OPCF	None	—	R	E/E	7	Death
4	51/M	Lung	Rapid	Pain	C1–3	P	Subtotal	OPCF	None	—	R + C	D/E	10	Death
5	70/M	Lung	Rapid	Pain	C1–2	HCA + P	Piecemeal	OPCF	None	R		E/E	13	Death
6	60/F	Lung	Rapid	Pain	C1	P	Piecemeal	OPCF	None	C	R	E/E	29	Alive
7	62/M	Lung	Rapid	Pain, dysphagia	C1	HCA + P	Piecemeal	OPCF	None	—	R + C	E/E	12	Death
8	46/M	Lung	Rapid	Pain	C2	HCA + P	Piecemeal	OPCF	Wound dehiscence dysphagia dysphonia	—	R + C	D/E	24	Death
9	42/M	Lung	Rapid	Pain	C2	HCA + P	Piecemeal	OPCF	None	C	R	E/E	18	Death
10	45/M	Lung	Rapid	Pain	C2	HCA + P	Piecemeal	OPCF	None	—	—	E/E	6	Death
11	60/F	Lung	Rapid	Pain	C2–3	HCA	Piecemeal	BCT	None	—		E/E	9	Death
12	62/F	Lung	Rapid	Pain	C2–3	P	Piecemeal	C1–4	None	—	C	E/E	19	Alive
13	50/M	Lung	Rapid	Pain	C2,4	P	Piecemeal	C2–4	None	C	R	E/E	6	Death
14	52/M	Lung	Rapid	Pain	C2–4	P	Piecemeal	C2–4	None	—	C	E/E	10	Alive
15	72/M	Lung	Rapid	Pain	C2	HCA + P	Piecemeal	BGT + C1–4	None	—		E/E	17	Alive
16	61/M	Nasopharyngeal	Rapid	Pain	C1	HCA + P	Piecemeal	OPCF	None	—	R	E/E	71	Death
17	56/M	Nasopharyngeal	Rapid	Pain	C1–2	P	Subtotal	OPCF	None	—	R	E/E	26	Death
18	63/M	Nasopharyngeal	Rapid	Pain	C1–3	P	Subtotal	OPCF	None	—		E/E	15	Alive
19	55/M	Nasopharyngeal	Rapid	None	C2	HCA + P	Piecemeal	OPCF	None	—	R	E/E	10	Death
20	27/M	Hepatic	Rapid	Pain	C2	HCA + P	Piecemeal	OPCF	None	—	R	E/E	36	Alive
21	64/F	Hepatic	Rapid	Pain	C2	HCA + P	Piecemeal	OPCF	None	—	R	E/E	39	Death
22	56/M	Hepatic	Rapid	Pain, dysphagia	C2	HCA + P	Piecemeal	OPCF	None	—	—	D/D	3	Death
23	54/F	Thyroid	Slow	Pain, numbness of the upper limbs	C2–4	P	Piecemeal	C1–5	None	—	R	E/E	78	Alive
24	70/M	Thyroid	Slow	Pain	C2	HCA + P	Piecemeal	BGT + OPCF	None	—		D/E	12	Death
25	45/F	Thyroid	Slow	Pain, dysphagia dysphonia	C1–2	HCA + P	Piecemeal	OPCF	None	—	R	E/E	60	Death
26	45/M	Renal	Moderate	Pain	C1	P	Piecemeal	OPCF	None	C	C	E/E	13	Death
27	58/M	Renal	Moderate	Pain	C1–2	HCA + P	Piecemeal	OPCF	None	R	R	E/E	18	Death
28	59/M	Renal	Moderate	Pain, numbness of the upper limbs	C1–4	P + HCA^a^	Subtotal	OPCF	None	R + C	R	E/E	6	Death
29	60/M	Osteosarcoma	Rapid	Weakness of the lower limbs	C2–4	P	Subtotal	C2–5	Hematoma, wound dehiscence	—	—	C/C	6	Death
30	8/M	Osteosarcoma	Rapid	Pain	C1–2	P	Subtotal	OPCF	None	C	—	E/E	4	Death
31	37/M	Osteosarcoma	Rapid	Pain, weakness of the upper limbs	C1–2	HCA + P	Piecemeal	OPCF	None	—	C	E/E	36	Death
32^#^	77/M	Prostatic	Slow	Pain	C2	HCA + P	Piecemeal	BCT + OPCF	Died of airway obstruction	—	—	D	0	Death
33	62/M	Prostatic	Slow	Pain	C1–2	P	Piecemeal	OPCF	None	—	R	D/E	22	Death
34	51/F	Breast	Slow	Pain	C1–2	HCA + P	Piecemeal	OPCF	Dysphagia, dysphonia	—	R	D/E	72	Death
35	49/F	Breast	Slow	Pain	C2–4	HCA + P	Piecemeal	BCT + C2–5	None	R + C	—	E/E	24	Alive
36	62/F	Basaloma	Slow	Pain	C2–3	HCA + P	Piecemeal	BGT + OPCF	None	—	R	E/E	10	Alive
37	80/M	Colon	Moderate	Pain	C1–2	HCA + P	Piecemeal	OPCF	Dyspnea	—		E/E	10	Death
38	69/M	Bladder	Rapid	Pain	C2–3	HCA + P	Piecemeal	BCT + OPCF	None	—	—	D/D	3	Death
39	30/M	Unknown	Rapid	Pain	C1–2	HCA + P	Piecemeal	OPCF	None	—	R + C	D/E	47	Death

*HCA* high anterior cervical approach, *P* posterior, *OPCF* occipitalcervical fusion, *BCT* bone cement and titanium, *BGT* bone graft and titanium, *R* radiotherapy, *C* chemotherapy

^#^Patient died of airway obstruction after operation

^a^The posterior approach was conducted first

Our indications for surgery were (1) severe occipital-cervical pain or significant fracture with atlantoaxial displacement; (2) failure of conservative treatment such as radiotherapy, chemotherapy, or targeted therapy; (3) occurrence of serious neurologic deficits; (4) to make histopathologic analysis of unknown primary tumor; (5) estimated survival time longer than 6 months; and (6) a tolerable general condition for surgery [4, 5].

Selections of surgical approaches and reconstructions generally depended on the location of the lesion as shown on pre-operative CT and MRI. A high cervical anterior approach (retropharyngeal) was adopted for patients with tumors that predominantly involved the C2 vertebral bodies or anterior arch and lateral mass of C1, while the posterior approach was performed in most patients for tumor resection and occipitocervical fusion. For patients treated with a combined approach, the anterior approach was done first (except case 28) to ensure the sufficient exposure of the anterior elements.

### Post-operative course

The trachea cannula was removed on the first day after operation in six patients and immediately after operation in the other patients. Adjuvant therapies were required according to the pathologies and pre-operative treatments (Table 1). All patients were required to stay in bed for about 3–4 weeks and wear the orthosis for at least 3 months. The follow-up evaluations were made regularly at 3, 6, and 12 months in the first year, then every 6 months or annually.

## Results

### Clinical findings

The patient demographics were summarized in Table 1. There were 29 males and 10 females (2.9:1), with a mean age of 55 (8–80) years. Neck pain, as the most common symptom, was complained by 37 (94.87%) of the 39 patients. Numbness or weakness of the upper limbs and pre-operative dysphagia were found only in 6 cases (Table 1). Only one patient (case 29) had the symptom of cord compression. The amount of solitary metastasis (C1 or C2), both metastasis (C1 and C2), and multiple metastasis involving the upper cervical spine were 15, 11, and 13 cases, respectively.

**Fig. 1:**
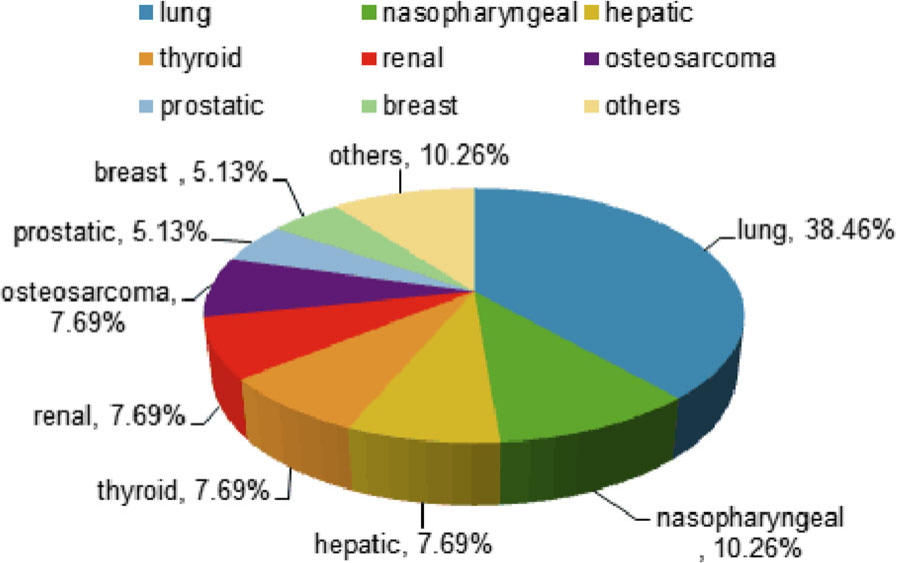
Pathologies proportion of the 39 atlantoaxial metastases

The primary sites of our cases were the lung in 15 (38.46%), nasopharynx in 4 (10.26%), and liver, thyroid, kidney, and bone (osteosarcoma) in 3 patients (7.69%), respectively (Fig. 1). Surprisingly, in 20 (51.3%) of the included patients, the symptoms of cervical metastases were the first presentations of primary malignant diseases, which are greatly different from the findings in previous studies [7, 11]. According to the Tomita scoring system, tumors of rapid growth group including lung cancer, nasopharyngeal carcinoma, hepatic cancer, osteosarcoma, and bladder carcinoma occupied about 69.23% (27/39). There were 7 patients in 2–3 points, 9 in 4–5 points, 11 in 6–7 points, and 11 in 8–10 points (Table 2). The mean pre-operative VAS was 6.46, and the pre-operative neurologic functions were grade D in 9 patients, E in 29 patients, and C in only one patient (Table 1).

**Table 2:** Survival time of different Tomita scoring groups

Tomita scoring	Cases	Mean survival (mos)	Median survival (mos)
2–3 points	7	29	60
4–5 points	9	33	47
6–7 points	11^a^	17	18
8–10 points	11	13	7

^a^The dead patient was excluded from this group

### Treatments and outcomes

With strict pre-operative assessment, all surgeries were completed successfully without serious intraoperative complications. Perioperative death occurred in one patient based on the World Health Organization (WHO) definition as death within 30 days after surgery. In broad terms, tumor resection was mainly conducted via the combined anterior retropharyngeal and posterior approach (66.67%), and the posterior approach was performed in 97.44% except one patient (case 11). Tumors were removed by piecemeal resection in 31 (79.49%) patients and subtotal resection in 8 (20.51%) patients. As to reconstruction, anterior reconstruction with autologous or allogeneic iliac crest bone grafts or bone cement and titanium mesh cages were performed in 6 patients, posterior reconstruction with occipitocervical fusion in 28 (71.79%) patients, and the remaining 5 patients were reconstructed with the combination of them.

All patients with pre-operative neck pain except the dead one experienced reduced or total relief of neck pain (97.30%), which is similar to the results in other studies [13, 19]. Paired *t* test of the pre-operative and post-operative VAS showed a great significance (*p* < 0.01) at 3 months follow-up with a post-operative VAS of 0.95. The neurologic function was recovered or improved after operation in all patients and did not show deterioration during the follow-up period (Table 1).

**Fig. 2:**
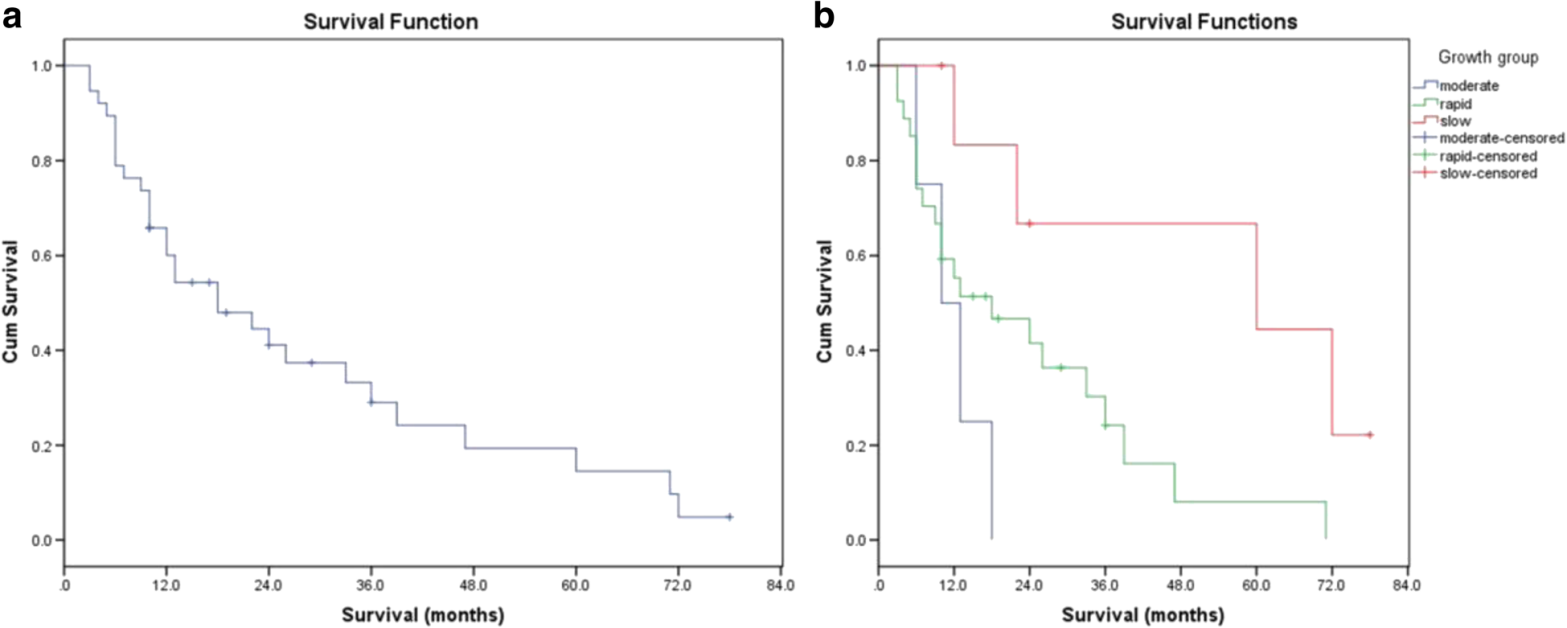
**a** Kaplan–Meier curve of 38 atlantoaxial metastases showed that the overall survival rates at 1, 2, and 3 years after surgery were 58.5, 40, and 28.3%, respectively. **b** Kaplan–Meier curves of different growth groups in atlantoaxial metastases showed that the median survival rates of slow, moderate, and rapid growth group were 60, 18, and 10 months, respectively

**Fig. 3:**
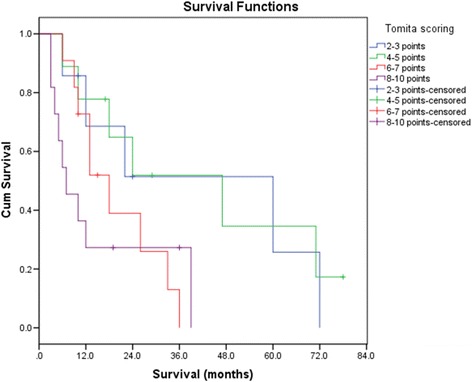
The survival time of different Tomita scoring groups

By the end of the follow-up period, 30 (76.92%) cases died mainly due to progression of systemic cancer, with no death due to local cord compression. Excluding the perioperative death, the survival time of others ranged from 3 to 138 months with a mean of 22 months. According to the Kaplan–Meier estimates (Fig. 2a), the median post-operative survival time was 18 months after surgery. The 1-, 2-, and 3-year overall survival (OS) was 58.5, 40, and 28.3%, respectively. The prognosis was relatively favorable in the slow-growth group, with a median survival of 60 months vs. 18 months in the rapid-growth group (Fig. 2b). Surprisingly, the prognosis in the moderate-growth group was poorer than that in the rapid-growth group, which probably due to the limited cases. The 1-year survival rate in patients with slow-growth primary tumors was 72.9% vs. 55.3% in patients with rapid-growth primary tumors. It is amazing that the 2-year survival rate was 41.5% in patients with rapid-growth primary tumors. The results of different Tomita scores showed a mean of 29 months in 2–3 points, 33 months in 4–5 points, 17 months in 6–7 points, and 13 months in 8–10 points, with an excellent median survival range of 7–60 months (Table 2, Fig. 3).

Generally, no more serious complication occurred in our series except that the patient who developed dyspnea after the tracheal extubation and died of airway obstruction at last. Another with dyspnea recovered well after emergent tracheotomy. There was still another patient who suffered posterior wound dehiscence and healed after debridement, while the other patient with wound dehiscence after the evacuation of hematoma failed to heal and died 6 months after operation due to the systemic tumor progression. No infection was noted in neither of them. Post-operative dysphagia and dysphonia occurred in two patients and vanished gradually without special intervention (Table 1). There was no other perioperative complications such as instrumentation failure, injury of the vertebral artery, neurologic deterioration, and cerebrospinal fluid leak in any of the remaining patients.

## Discussion

### Significance of surgical treatment

Atlantoaxial metastasis is a rare clinical occurrence, accounting for about 0.5% of all spinal metastases. Up to now, the treatment choices of such metastases are controversial, especially in terms of the surgical approaches, reconstruction, and the extent. Here, we mainly aimed to investigate the surgical decisions and outcomes so as to provide more references for the treatment of this special and complex metastasis through sharing our experience of treating upper cervical spine metastases.

**Fig. 4:**
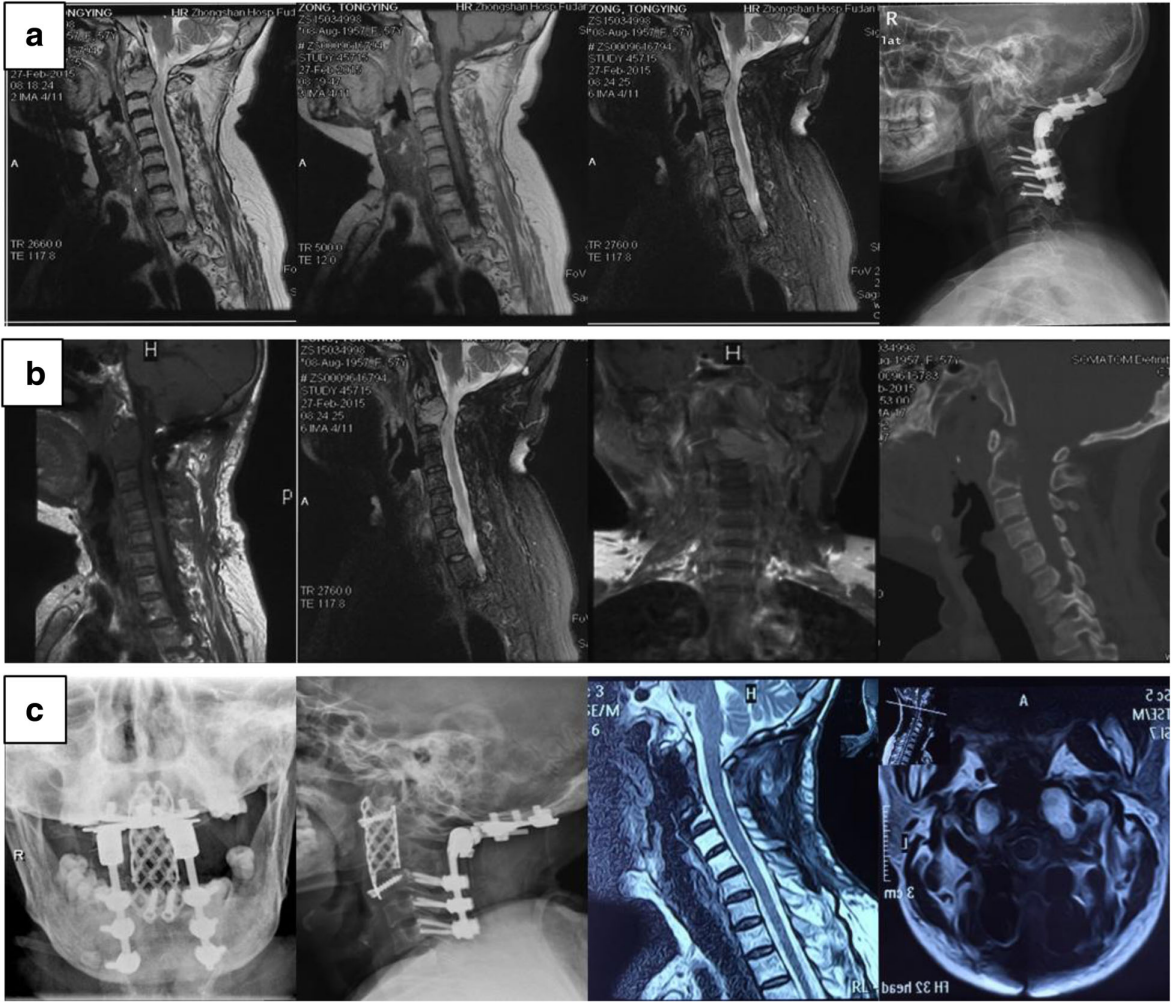
One case with C2 metastasis (thyroid cancer) suffered serious pain for half a year. **a** The posterior occipitalcervical fixation was conducted only when he was first adopted into another hospital. **b** He came to our center for unrelieved continuous neck pain. We performed the tumor resection through an anterior-posterior approach, followed by the spine reconstruction without complication. **c** The last follow-up at 11 months without recurrence and discomfort

Patients with atlantoaxial metastases may remain asymptomatic for a long time because of the wide diameter of the upper cervical spinal canal [16]. Pain, which can be intolerable in some cases, is the most common pre-operative symptom [1, 7–9, 11, 13–15], as was in our study (94.87%). Instability is the main reason for pain, which usually cannot be relieved completely by radiotherapy alone [17]. Instead of surgical resection, relative conservative therapy used to be the main choice for atlantoaxial metastasis primarily because of the limited life expectancy, high risks of surgery, and inadequacy of the techniques available [6–11]. Of course, once the failure of conservative therapies, progression of tumors, or limited relief of symptoms occur, more serious complications even death were inevitable and surgeries may be required at last [9, 11, 13, 16, 17]. In our series, though some complications may occur, the anterior surgery is recommended since the failure possibility of conservative therapies and the relatively low rate of complications. Here, eight of the 39 patients were admitted to our center for the ineffective relief of the symptoms by adjuvant therapies. Therefore, we recommend to proceeding relatively aggressive tumor resection (Fig. 4) to reduce the low mortality since the advances of surgical techniques today and the significant low rate of complications.

Pain relief is usually the earliest outcome in the treatment of upper cervical metastasis, as reported in previous studies [7–11]. Similarly, pain relief in our presentation was extremely excellent (100%) with a mean VAS of 0.95 after operation vs. 6.45 before operation (*p* < 0.01), which improves patients’ quality of life greatly. Survival is another important consideration in deciding whether to perform surgery or not and the extent of surgery for upper cervical metastasis. More reports [11, 13, 16, 20] have showed that surgery can help achieve better prognosis and survival than palliative or conservative interventions for cervical and other spinal metastases.

In our series, the average survival time of the 38 (except the perioperative death) patients was 22 months, longer than that of other studies of cervical metastases with a mean survival of 6–19 months [1, 4, 7, 8, 10, 19, 21–23]. Yet, Kirchner et al. [5] reviewed all the metastases to the cranio-cervical junction with a mean survival time of 6.44 months. In addition, the median survival time in our series was 18 months, and the 1- and 2-year OS was 58.5 and 40%, respectively, which were better than the 1-year OS of 58% and 2-year OS of 21% reported by Heidecke et al. [22], and the 1-year OS of 33% reported by Fourney et al. [10]. Although the Tomita scores were high in about 50% of our cases, the overall outcome was satisfactory as represented by a mean survival rate of 13 months. It was obvious that surgical treatment benefit patients of atlantoaxial metastasis greatly in spite of the high Tomita scores.

### Surgical approach

There are controversies over the selection of surgical approaches for upper cervical spine tumors. Ideally, the optimal surgical approach should be the one that can provide access to all components for convenient resection and reconstruction without causing injury to the vital structures. In general, the tumor location, the extent of location, the patient’s general condition, potential complications, and surgical experience are critical factors affecting the decision on the selection of surgical modalities. In previous studies, the posterior approach alone was performed mainly for the purpose of regional stabilization [5–8, 11]. Various approaches can give access to the atlantoaxial lesions, including the transmandibular, transoral, anterolateral, posterolateral, high anterior cervical, combined pre-vascular and retrovascular extraoral, posterior, and the combined anterior and posterior approach [4, 24–26].

Similar to tumors of the subaxial cervical spine, atlantoaxial metastases generally involve the anterior elements (WBB, section 4–9). The anterior approach for resection of upper cervical tumors in this location is essential in most cases but was infrequently used in atlantoaxial metastases before [4–8, 11, 26]. Maxillotomy, transmandibular, and transoral approach were seldom used in atlantoaxial metastases since complications such as large trauma, infections, and delayed union are the most common problems [27, 28]. The high anterior cervical approach avoids the infection risk of the transmandibular and transoral approach, and also allows for enough exposure to tumors of lateral mass, anterior arch, odontoid process, and C2 vertebra. In this study, we employed the high anterior cervical approach for all the anterior resection. Our practice and other studies [4, 25, 29] have demonstrated that the high anterior cervical approach is effective to perform tumor resection and reconstruction in this region.

In our study, the posterior approaches with or without occipitocervical fusion were performed in 31 and 7 cases respectively (Table 1). The posterior approach, especially occipitocervical fixation, seems to be inevitable for upper cervical spine tumors to keep the stabilization in this region [5, 8, 10, 14]. Certainly, posterior fixation alone without the occiput can also be considered for C2 alone tumors [30, 31].

Although en bloc resection is now recommended as the optimal therapeutic option for spine tumor [32], it is difficult to be performed in the upper cervical spine. For metastases of the upper cervical spine, we prevailingly performed piecemeal resection for most of the patients due to the complexed anatomies. At last follow up, no further neurologic deterioration was observed in our patients. It was obvious that total piecemeal resection plays an important and useful role for atlantoaxial metastasis.

### Reconstructions

Reconstruction after atlantoaxial tumor resection is another problem in terms of the method and material [30, 31]. For atlantoaxial lesions, early surgeons performed anterior reconstructions with bone grafts fixed to anterior arch or clivus or combination of bone grafts, plates, and screws, but neither of them was sufficient [33]. In recent years, the titanium mesh cage with or without plate modified types and prosthesis have been employed to gain solid reconstruction in this region [30, 34]. For lesions involving the anterior structures of the atlas, reconstruction seems to be more difficult and insufficient. Given this situation, we performed the surgeries without anterior reconstruction in this case for the possible complications induced by failure of internal fixation. In our opinion which was proved by practices, removal of the anterior arch and one of the lateral mass will not affect the stability significantly with the help of occipitocervical fusion. In addition, for patients treated with a combined approach, we preferred to perform the anterior approach first (except case 28) in case that occipitalcervical fusion may prevent from sufficient exposure for resection and reconstruction of the anterior.

### Pathology

As shown in Fig. 1, the most common atlantoaxial metastasis was seen in lung cancer, followed by nasopharyngeal, hepatic, thyroid, renal cancer, and osteosarcoma. Unlike breast cancer, metastases in lung and prostate cancer mainly involve cervical or upper cervical spine [4, 7, 9–11, 22], and the rapid-growth tumors rank the most (69.23%). As a result, the prognosis varied in different growth groups as mentioned above (Fig. 2). This reminded us that relative aggressive surgical resection was worthy for upper cervical metastases, especially for the slow-growth group.

### Complications

Complications of atlantoaxial tumors treatments are usually weighted carefully in decision-making of surgical or non-surgical treatment, Complications associated to the surgical treatments including injury of the spinal cord, hemorrhage, dyspnea, wound infection, and fixation failure are various and deadly [8–10]. Others include damage to the superior laryngeal and hypoglossal nerves, wound dehiscence, and cerebrospinal fluid leakage. It was reported [35, 36] that the overall rate of complications was as high as 14–34% in the surgical treatment of cervical spine tumors. The rate of complications associated with surgeries in this study was as low as 12.82% (5/39) though the combined approach was conducted in most of the cases (Table 1). In more detail, complications associated with the anterior approach were dysphagia and dysphonia in two patients, airway obstruction and tracheotomy in one patient, respectively, which was a relative low rate (4/39, 10.26%). The most serious complication in our series was dyspnea in one patient who died of airway obstruction. Hence, tracheal extubation should be done after careful assessment of the breathing status and meticulous preparation of tracheotomy. Although complications are unavoidable in some cases, efforts should be made to reduce complications via careful pre-operative evaluation, careful and gentle operation, and post-operative close observation. To sum up, aggressive surgery is recommended for atlantoaxial metastases since the advance in surgical techniques and low incidence of complications and reliable outcomes.

### Adjuvant therapy

Adjuvant therapies such as radiotherapy and chemotherapy were mainly used in atlantoaxial metastases, while the efficacy was limited [17]. In our study, pre-operative adjuvant therapies were performed in 10 patients (25.64%) before surgery. The timeframe of adjuvant therapies was determined by the tumor types as well as the urgency of symptoms. Generally speaking, according to our experience, adjuvant therapies such as radiotherapy, chemotherapy as well as targeted therapy were carried out after the surgical intervention rather than before for the immediate stabilization, pain relief, and the prevention of possible neurologic deterioration as well as the possible wound healing problem. Though all patients were advised to receive post-operative adjuvant therapy according to their pathologies and pre-operative treatments, unfortunately, the compliance of receiving adjuvant therapies was not very satisfactory in this study (Table 1), which may in turn reveal the surgical efficacy to some extent.

In summary, our experience and practice suggest that more aggressive surgery should be considered in the treatment of atlantoaxial metastases since the critical function of atlantoaxil spine for people’s life. The main goals of surgical intervention are to (a) relieve pain, (b) restore immediate spinal stability, (c) prevent neurologic deterioration, (d) obtain histological diagnosis, and (e) increase the obedience for further adjuvant therapies. Of course, some limitations of this study should be listed. First, this study is a retrospective one, which is not as evident as a randomized controlled trial, through it is greatly difficult to carry out. Second, the amount of patients collected in this study is limited, and studies of more cases are needed. In addition, the effect of adjuvant therapy in this study was unable to be evaluated exactly. In all, studies including more cases, randomized controlled trial and multiple-factor analysis are urgently required to gain the treatment guidelines of upper cervical spine metastases.

## Conclusions

Metastatic involvement of the upper cervical spine is rare and intractable. Pain is the most common symptom. Relative radical surgical resection with effective stabilization can achieve satisfactory outcomes with a low rate of mortality and complication. Together with adjuvant management, surgical treatment of atlantoaxial metastases can not only relieve regional pain, restore, or improve neurologic function but also stabilize the quality of life and prolong the survival time of such patients.
